# Supplementary material for: Identifying SARS-CoV-2 antiviral compounds by screening for small molecule inhibitors of Nsp5 main protease
Source: Biochem J. 2021 Jul 2;478(13):2499–515. doi: 10.1042/BCJ20210197 (PMC8286836; doi:10.1042/BCJ20210197)

Supplemental Material

Figure S1: Testing reaction buffers for high-throughput screening

A - B) Increase in fluorescence over time after initiation of nsp5 protease FRET-based assay onto either DMSO or water via hand pipetting (A) or via an automated liquid handling robot (B). Experiments were done in triplicate.

C) The nsp5 protease FRET-based assay was monitored for increase in fluorescence over time in standard buffer (50 mM Hepes-KOH pH 7.6, 2 mM DTT and 1 mM EDTA) onto either DMSO or water. 10% glycerol or 0.02% Tween are added to the standard reaction buffer which is dispensed onto either DMSO or water.

Figure S2: IC_{50} values and related figures of all hits

A-D) *In vitro* activity of nsp5 was measured over a wide-range concentrations of different inhibitors using the FRET-based assay. A dose response curve for an IC_{50} value and Hill coefficients (nH) was determined by non-linear regression (shown in black) for all drugs where possible. Drugs are sorted into different stages of validation. All FRET-based IC_{50} values are given as mean ± SEM, n=3. Data on the graphs is plotted as mean and error bars represent SD. For select compounds a nsp9 gel-based assay was done at a wide range of drug concentrations.

(D) Calpain Inhibitor I, Z-VAD-FMK, Z-DEVD-FMK and Z-IETD-FMK were all validated as specific nsp5 inhibitors. FRET-based assay dose dependent curves are shown with IC_{50} values determined by non-linear regression with normalised data alongside relevant chemical structures. FRET-based IC_{50} values are given as mean ± SEM, n=3. Data on the graphs is plotted as mean and error bars represent SD..

Figure S3: Enzyme characterisation in different reducing environments

A) Graph of K_M determination. Initial reaction rates at a range of concentrations were plotted against substrate concentration to obtain values for K_M using different reducing reagents in the buffer (no reducing agent, DTT, GSH).

Figure S4: Antiviral activity of HTS hits in combination with remdesivir against SARS-CoV-2 in Vero E6 cells

A) Dose response curves for the validated hits (Calpain Inhibitor I and Z-VAD-FMK) in combination with remdesivir in a viral infectivity assay in Vero E6 cells. Cell viability values represent the area of cells stained with DRAQ7 DNA dye. Viral infection values were measured as the area of viral plaques visualised by immunofluorescent staining of viral nucleocapsid protein. Data is normalised to DMSO only treated control wells (100 %) and plotted as mean and standard deviation (SD), n=3. EC_{50} values were determined by non-linear regression and values are given as mean ± SEM.

B) Representative images for the SARS-CoV-2 viral infectivity assay in Vero E6 cells, for Calpain Inhibitor I and Z-VAD-FMK in combination with remdesivir. Representative wells show Vero E6 cells stained for DNA using DRAQ7 (top panel, labelled cells), and viral N protein immunofluorescence (lower panel, labelled virus).

Figure S5: Antiviral activity of improved FMK inhibitor in combination with remdesivir against SARS-CoV-2 in Vero E6 cells

A) Dose response curves for the custom inhibitor Z-AVLD-FMK in combination with remdesivir in a viral infectivity assay in Vero E6 cells. Cell viability values represent the area of cells stained with DRAQ7 DNA dye. Viral infection values were measured as the area of viral plaques visualised by immunofluorescent staining of viral nucleocapsid protein. Data is normalised to DMSO only treated control wells (100%) and plotted as mean and SD, n=3. EC_{50} values were determined by non-linear regression and values are given as mean ± SEM.

B) Representative images for the SARS-CoV-2 viral infectivity assay in Vero E6 cells, for Z-AVLD-FMK in combination with remdesivir. Representative wells show Vero E6 cells stained for DNA using DRAQ7 (top panel, labelled cells), and viral N protein immunofluorescence (lower panel, labelled virus).

Figure S1: Testing reaction buffers for high-throughput screening

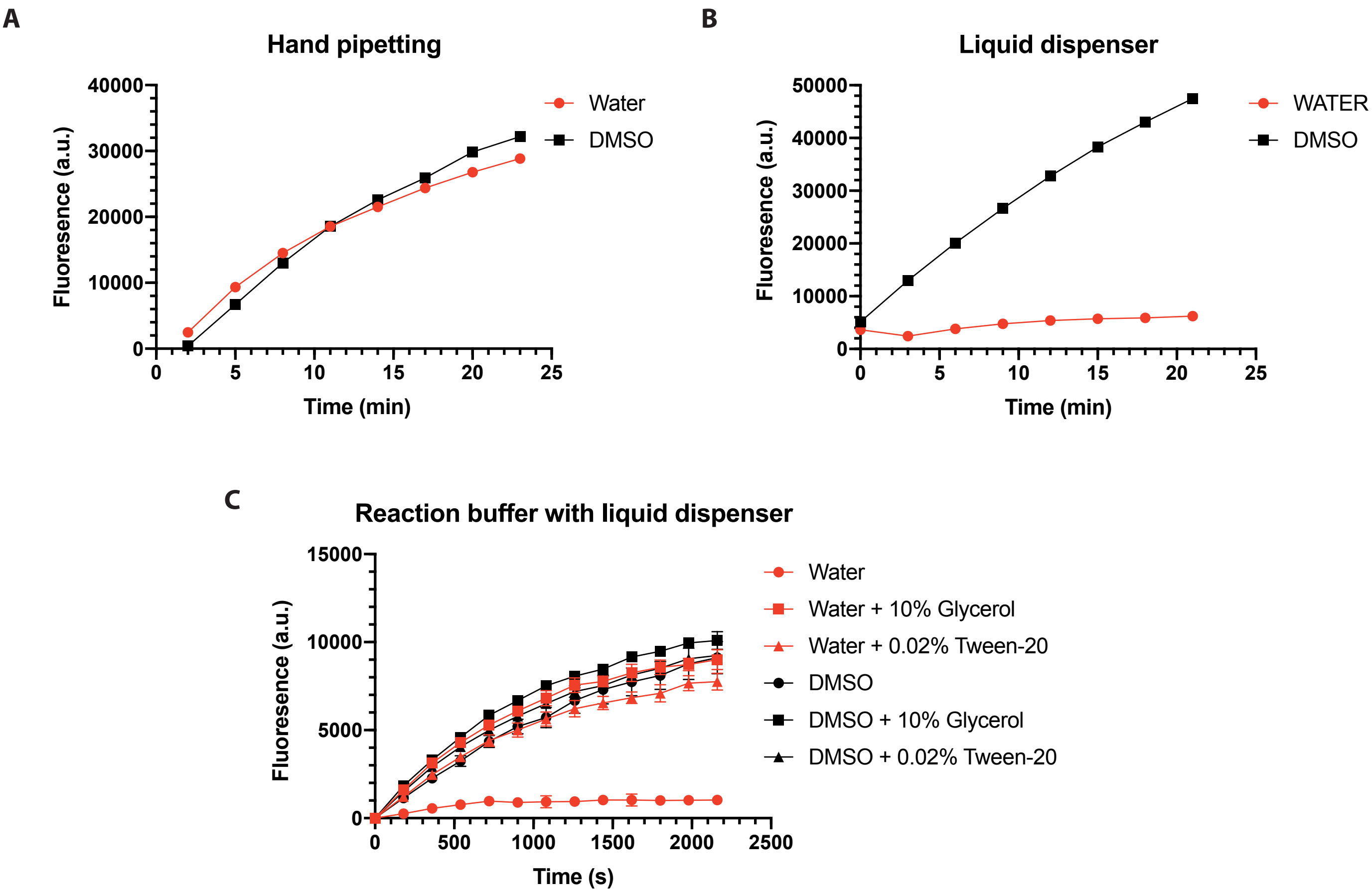

Figure S2: IC_{50} values and non-inhibitors

A Did **not** inhibit in FRET-based assay:

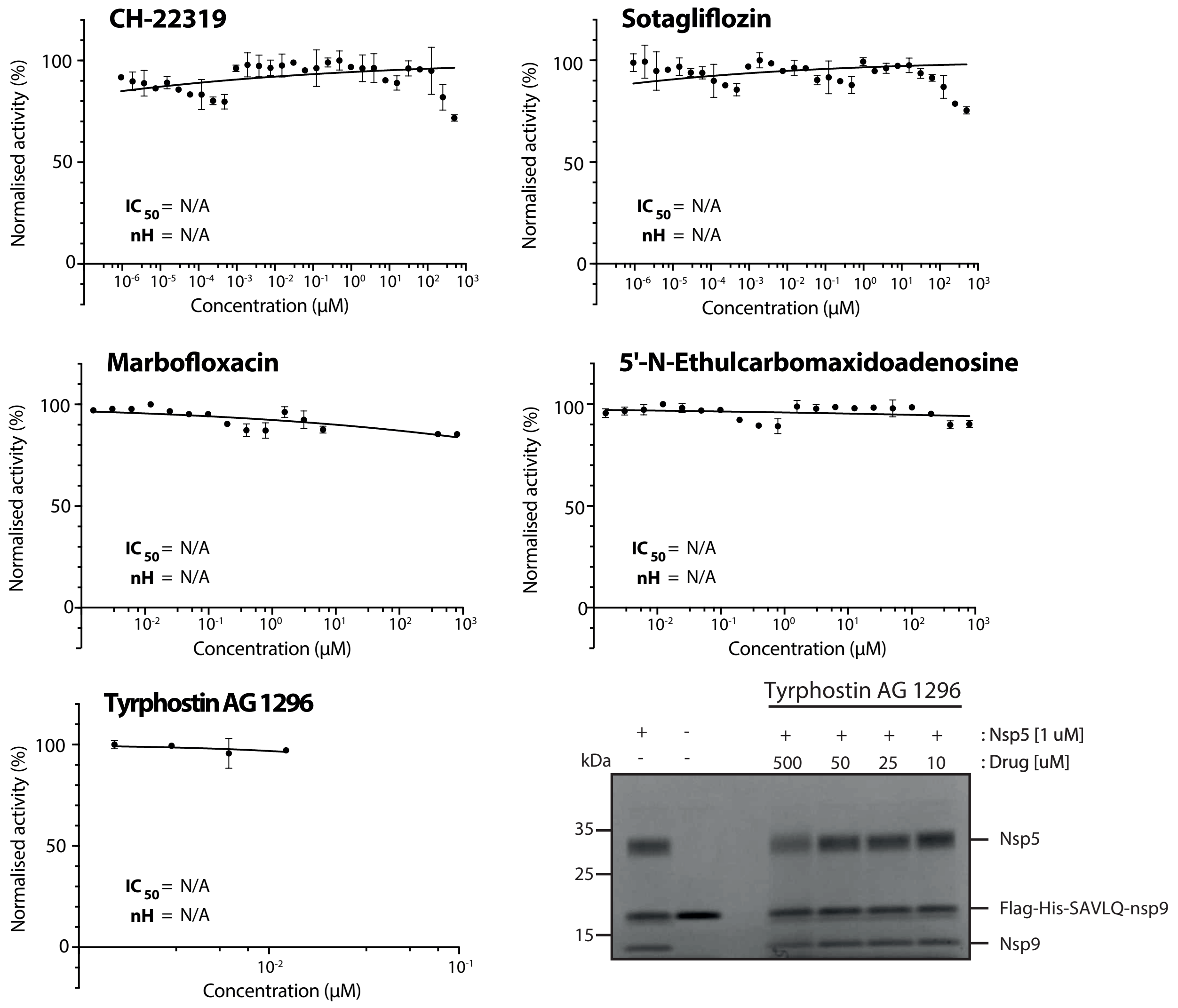

B Did **not** inhibit in gel-based assay:

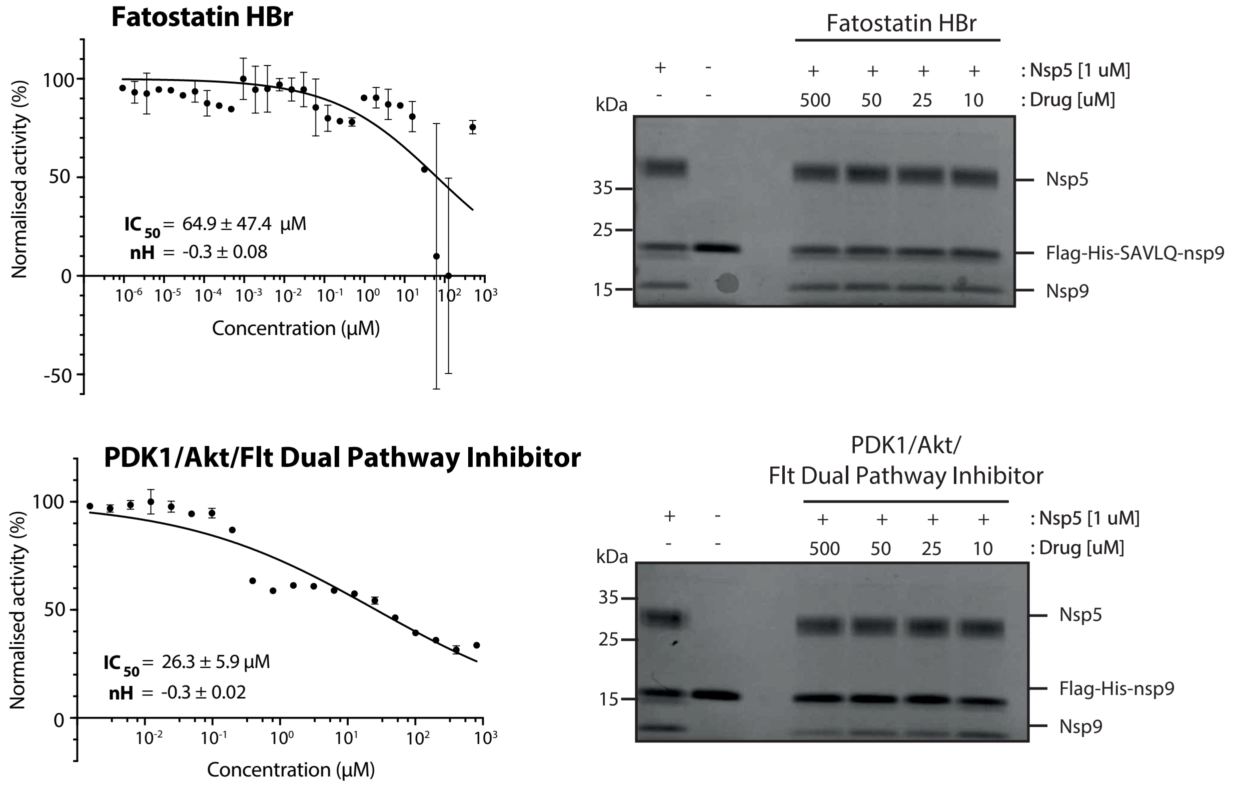

C Inhibited thrombin in primary screen buffer:

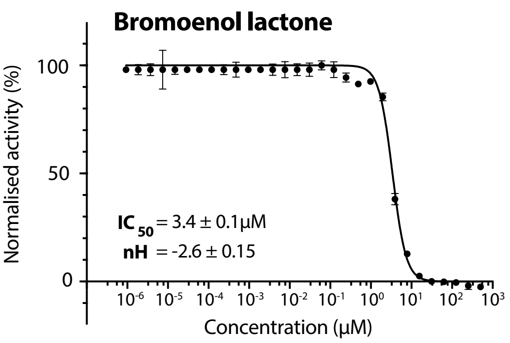

D Final Nsp5 Inhibitors:

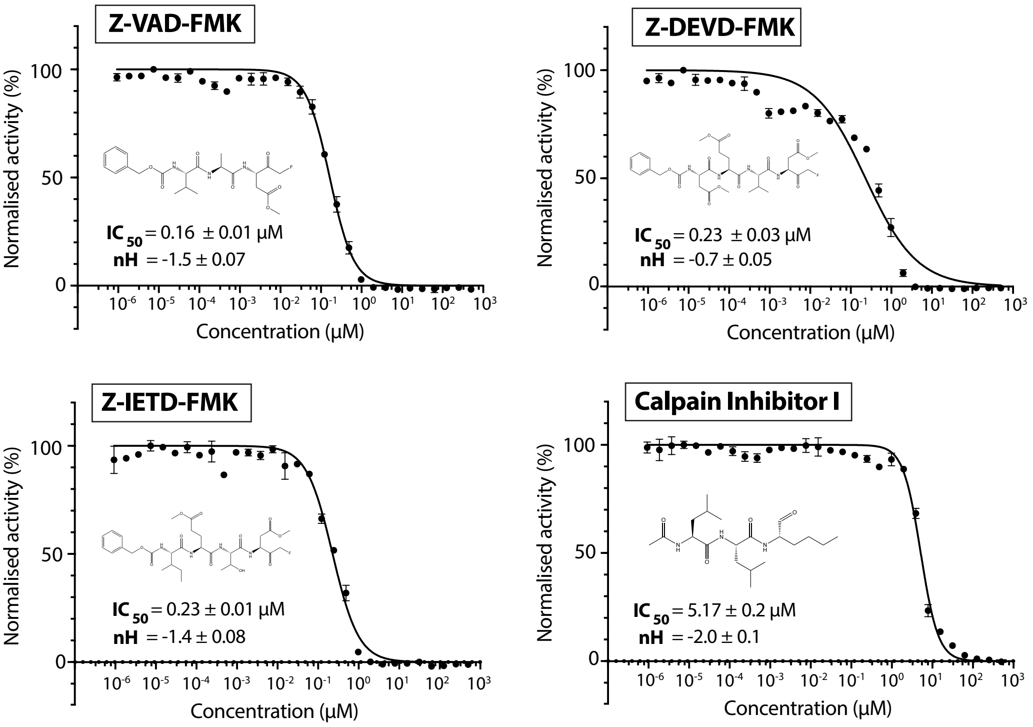

Figure S3: Nsp5 enzymatic characterisation in different reducing environments

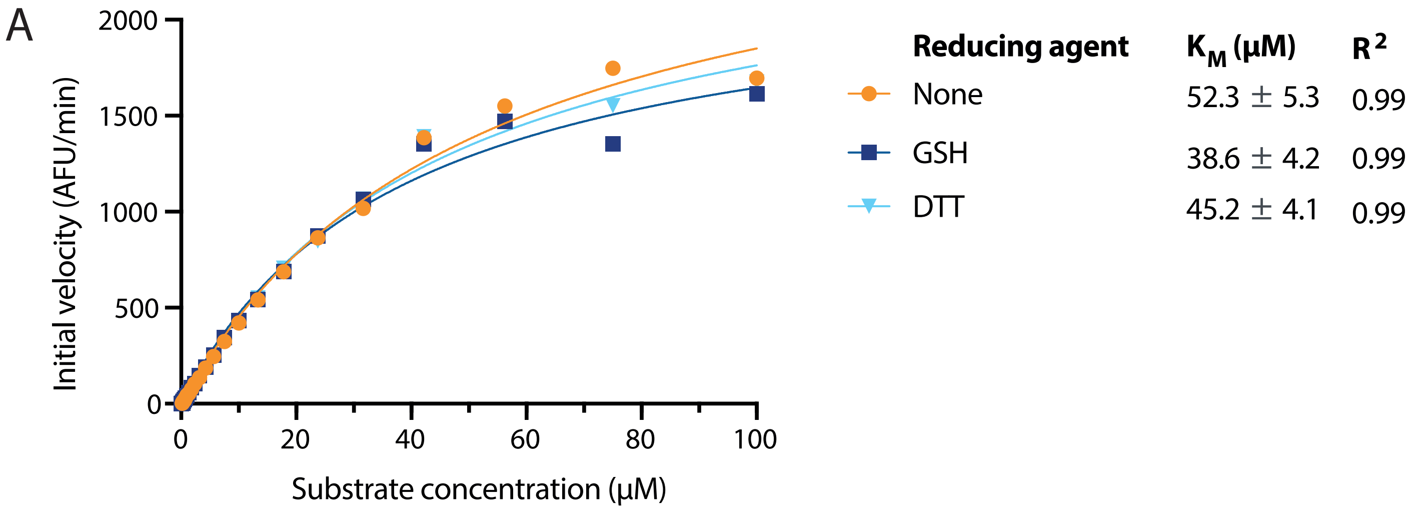

Figure S4: Antiviral activity of drugs in combination with remdesivir in Vero E6 cells

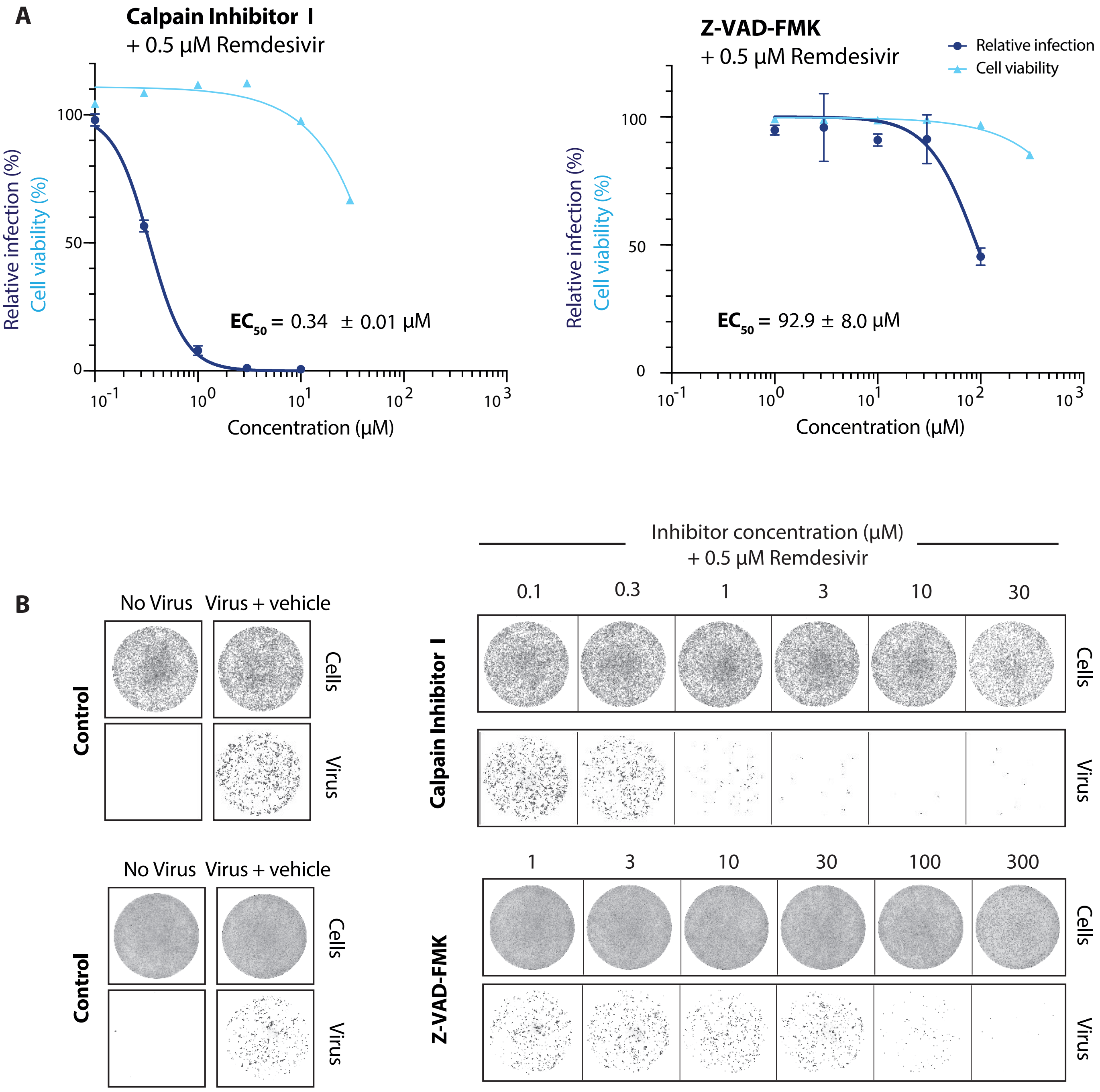

Figure S5: Antiviral activity of improved FMK inhibitor in combination with remdesivir in Vero E6 cells

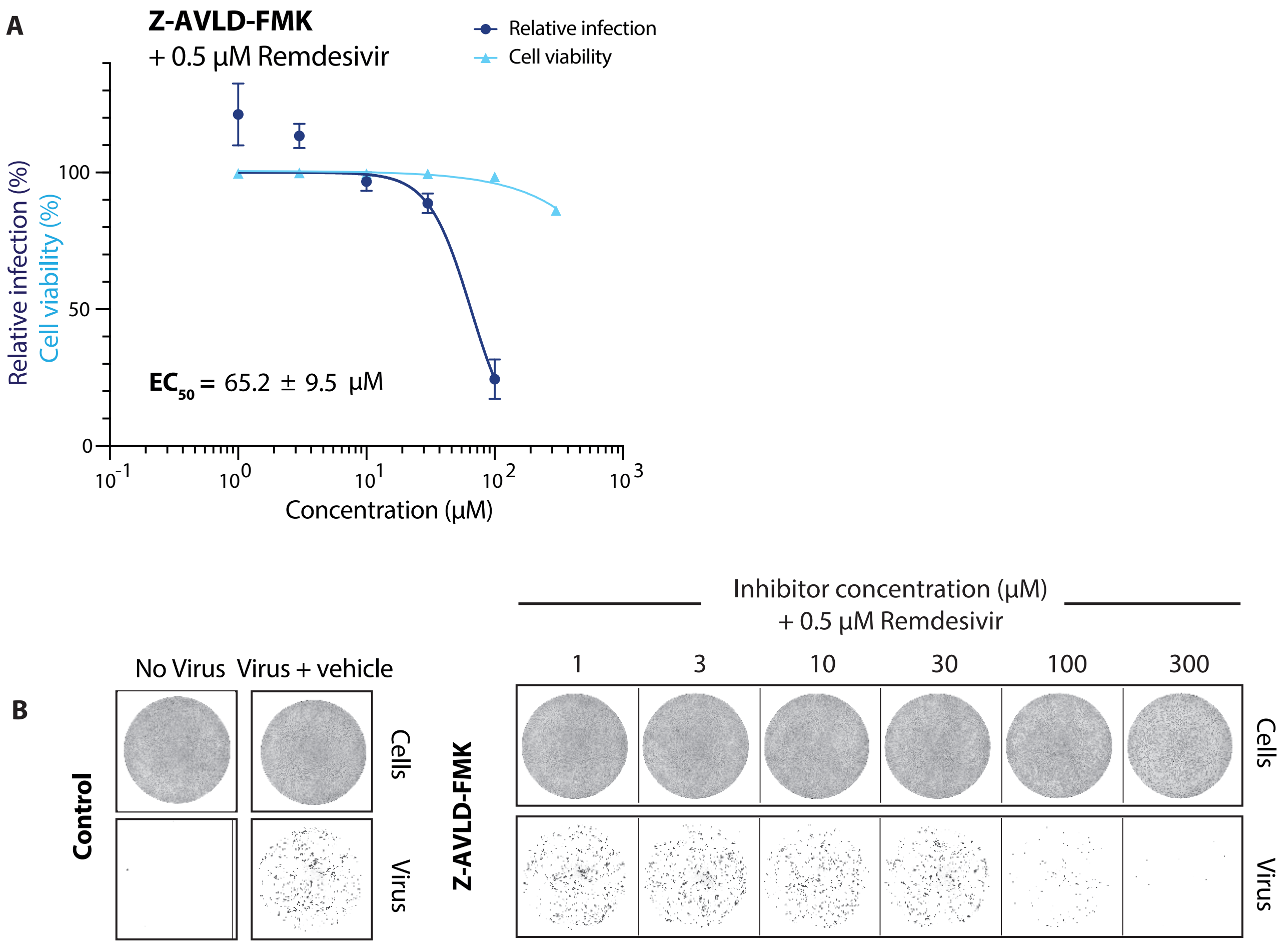

Supplement: Supplementary Figures S1-S5 [file BCJ-478-2499-s1.pdf]
